# DeRitis ratio is associated with unfavorable prognosis in ACS patients

**DOI:** 10.3389/fcvm.2026.1690027

**Published:** 2026-03-17

**Authors:** Yi Fang, Chensong Zhang, Haiwei Liu, Yang Li, Miaohan Qiu, Kai Xu

**Affiliations:** 1 State Key Laboratory of Frigid Zone Cardiovascular Disease, Cardiovascular Research Institute and Department of Cardiology, General Hospital of Northern Theater Command, Shenyang, China; 2 Department of Cardiology, Shenyang Ninth People’s Hospital, Shenyang, China

**Keywords:** ACS, AST/ALT, DeRitis ratio, survival analysis, unfavorable prognosis

## Abstract

**Background:**

The aspartate aminotransferase (AST) to alanine aminotransferase (ALT) ratio, commonly referred to as the DeRitis ratio, serves as an indicator of liver disease severity and has been linked to several cardiovascular risk factors. This study aimed to explore the relationship between the DeRitis ratio (AST/ALT) and long-term adverse outcomes in patients diagnosed with acute coronary syndrome (ACS).

**Methods:**

We selected 8,429 patients with ACS from the OPT-CAD database who were admitted between 2012 and 2014 and had complete AST and ALT measurements. Following propensity score matching (PSM), 5,680 patients were included in the final analysis. A retrospective survival analysis was conducted to evaluate the relationship between the DeRitis ratio and the occurrence of net adverse clinical events (NACE) within 5 years post-discharge. The primary outcome was NACE, defined as a composite of all-cause mortality, stroke, non-fatal myocardial infarction (MI), and major bleeding [Bleeding Academic Research Consortium (BARC) types 2–5].

**Results:**

Following PSM, the cumulative incidence of NACE within 5 years was significantly higher in the group with a DeRitis ratio ≥1 compared to the group with a DeRitis ratio <1 (HR: 1.21; 95% CI: 1.05–1.38; *P* = 0.0071). After adjusting for confounding factors using Cox multivariable analysis, the association persisted as significant (HR: 1.21; 95% CI: 1.06–1.39; *P* = 0.006). Subgroup analysis indicated that the high-ratio group had a significantly increased risk of major bleeding (HR: 1.27; 95% CI: 1.01–1.59; *P* = 0.037). However, the cumulative risk of all-cause death showed a strong trend towards increase but did not reach statistical significance (HR: 1.23; 95% CI: 0.99–1.51; *P* = 0.052). There were no significant differences between the groups in the cumulative risks of non-fatal myocardial infarction (MI) (HR: 1.22; 95% CI: 0.89–1.67; *P* = 0.21), or stroke (HR: 1.01; 95% CI: 0.78–1.32; *P* = 0.92).

**Conclusion:**

The DeRitis ratio ≥1 is significantly associated with elevated risk of NACE during the 5-year post-discharge period in ACS patients. Additionally, an elevated DeRitis ratio is correlated with a higher incidence of major bleeding events.

## Background

Coronary artery disease (CAD) ranks as the top cause of mortality globally (1). Since the year 2000, it has shown the most significant rise in mortality rates worldwide (2). Finding dependable biomarkers to anticipate negative outcomes in CAD is still a primary area of research. These indicators could help in early detection and minimize the chances of adverse prognoses.

Liver function relies on essential enzymes, such as AST and ALT, to operate efficiently. Their ratio, known as the DeRitis ratio (AST/ALT), was originally introduced as a component of liver function tests (3) and has been widely used to assess alcohol-related and non-alcoholic liver damage (4, 5). In mild hepatitis, for example, cellular and mitochondrial injury are generally limited, and the DeRitis ratio typically remains below 1; an elevated ratio often suggests more severe parenchymal liver damage (6). Beyond hepatology, both enzymes have also been studied for their prognostic value in CHD and heart failure (7, 8). This is partly because ALT is primarily derived from hepatocytes, whereas AST has multiple sources, including the heart, red blood cells, and other tissues (9).

An elevated DeRitis ratio may reflect underlying pathological processes such as hepatic steatosis, systemic inflammation, or insulin resistance (10, 11), which are themselves linked to adverse cardiovascular outcomes. Thus, this ratio may serve as an integrative marker of systemic metabolic and inflammatory burden. The ability of specific liver enzymes to accurately predict negative heart-related events is restricted, and existing research has shown inconsistent findings. For example, the Framingham Offspring Study indicated that higher liver enzyme levels might be linked to a heightened risk of cardiovascular diseases and metabolic disorders (12). In contrast, various other investigations observed no meaningful connection to negative outcomes (13).Considering the fluctuations of AST and ALT in predicting diseases, utilizing the DeRitis ratio could offer a more consistent and comprehensive biomarker for monitoring CAD development. Liver fibrosis scores that include the DeRitis ratio have indeed been developed as new methods to forecast recurrent cardiovascular incidents in secondary prevention (14).While earlier studies have suggested the possible usefulness of the DeRitis ratio for forecasting cardiovascular events in the general public (15), its connection with long-term net adverse clinical events (NACE) in individuals with acute coronary syndrome (ACS) has yet to be investigated.

## Method

### Study population

This retrospective examination utilized information from the Optimal Antiplatelet Therapy for Chinese Patients with Coronary Artery Disease (OPT-CAD) study. This multicenter prospective registry (NCT01735305) included 14,032 CAD patients collected from 107 locations in China from January 2012 to March 2014. The enrollment period was predefined by the OPT-CAD study protocol to ensure a standardized cohort with complete long-term follow-up. While percutaneous coronary intervention (PCI) techniques and guidelines evolved during this time, the primary objective of this analysis was to evaluate the prognostic value of a biochemical marker (DeRitis ratio), which is largely independent of specific procedural advancements. Throughout the study, every participant was administered a minimum of one antiplatelet medication.

In this group, we incorporated 8,429 ACS patients with full ALT and AST test results. The patients were separated into two categories using a DeRitis ratio cutoff of 1. This cutoff is widely used in clinical hepatology to indicate more significant hepatocellular damage and is commonly applied in cardiovascular research to facilitate comparison with existing literature (3, 15). Propensity score matching (PSM) was utilized to equalize initial characteristics in order to reduce possible confounding variables. Following the matching process, 5,680 patients were kept for the concluding analysis. During the 5-year follow-up period, 192patients (1.4%) were lost to follow-up and were censored at the time of last contact in the survival analysis.

### Study design

The eligibility requirements for the OPT-CAD study have been detailed in an earlier publication (16). On the first visit, researchers proactively filled out a case report form for every patient, documenting demographic details, medical history, presenting symptoms, lab findings, treatment plans, and results during hospitalization. All variable definitions and clinical diagnoses conformed to established criteria.

**Figure 1:**
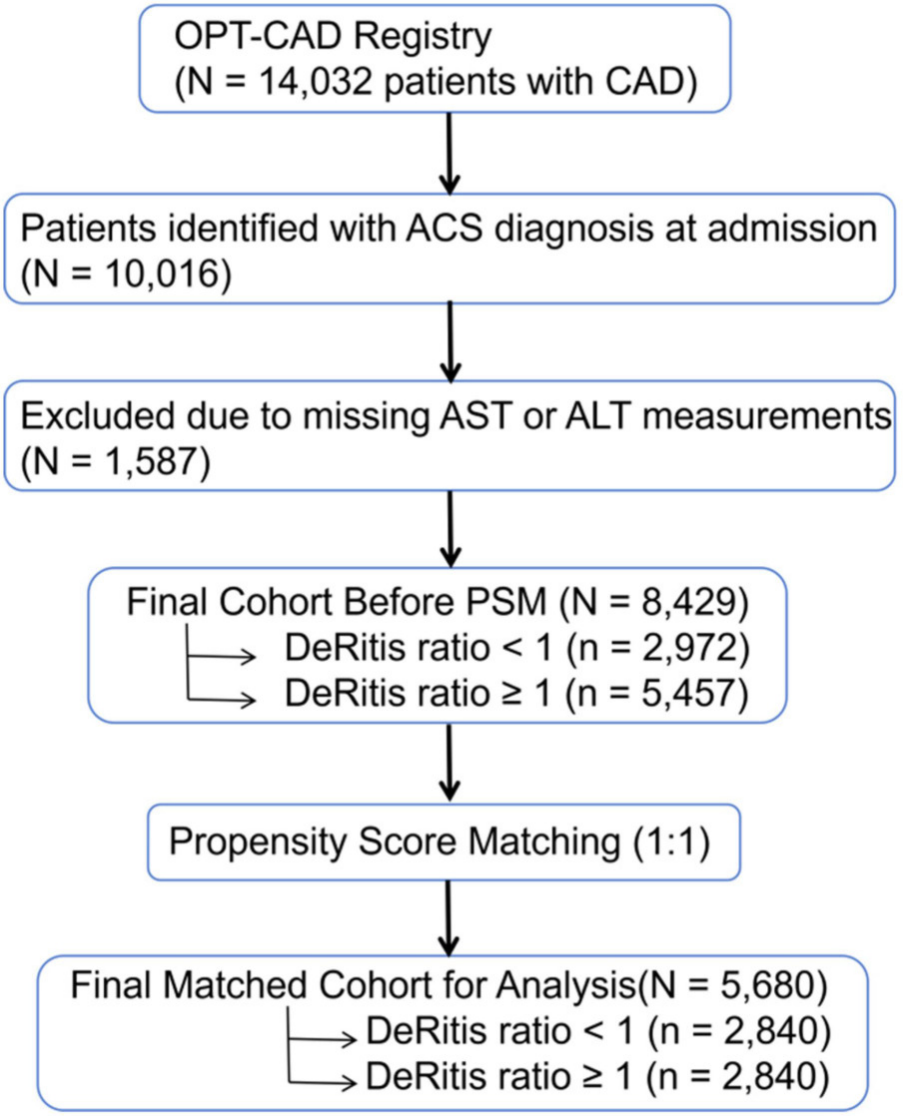
Patient selection flowchart. ACS, acute coronary syndrome; AST, aspartate aminotransferase; ALT, alanine aminotransferase; PSM, propensity score matching.

From the OPT-CAD cohort, we identified a cohort of 10,016 patients who were diagnosed with ACS at the time of admission. The DeRitis ratio is determined by dividing AST levels by ALT levels. Individuals lacking AST or ALT values were omitted from the analysis, as no imputation was performed, leading to the exclusion of 1,587 patients. Consequently, the starting group comprised 8,429 patients who met the eligibility criteria. After utilizing PSM to equilibrate baseline covariates, a total of 5,680 patients remained for the final examination. Figure 1 presents the patient selection flowchart.

The main goal was to track the incidence of NACE within five years after discharge, characterized as a combination including all-cause mortality, stroke, non-fatal myocardial infarction (MI), and significant bleeding events [Bleeding Academic Research Consortium (BARC) types 2–5] (17). Secondary objectives encompassed the individual elements that make up the NACE composite.

### Measurements

Three more parameters need to be calculated for this analysis. To calculate BMI, divide an individual's weight in kilograms by the square of their height measured in meters. The estimated glomerular filtration rate (eGFR) was determined using the simplified Modification of Diet in Renal Disease (MDRD) study equation (18):eGFR (ml/min/1.73 m^2^) = 186 × (Serum Creatinine in mg/dl)^−1·1^⁵⁴ × (Age)^−0·2^⁰^3^ × (0.742 if female). The DeRitis ratio refers to the comparison between the levels of AST and ALT enzymes.

### Statistical analysis

All assessments were carried out using R software, version 4.0.5. (R Foundation for Statistical Computing, Vienna, Austria). Key packages including “MatchIt” and “survival” were employed. Categorical variables are shown in numbers and percentages, whereas continuous variables are detailed using means with 95% confidence intervals (CIs) or mean ± standard deviation, depending on the situation. A *t*-test was used to analyze continuous variables with a normal distribution for group comparison. We utilized Pearson's chi-square test for analyzing differences among categorical variables. The Mann–Whitney *U*-test was utilized to analyze data that deviated from a normal distribution. In order to mitigate potential confounding factors, logistic regression analysis was employed to pinpoint covariates for incorporation into propensity score matching (PSM).Propensity score matching (PSM) was utilized to equalize initial characteristics across the groups being compared. Differences in survival between patients with a DeRitis ratio of 1 or higher and those with a ratio below 1 were assessed through Kaplan–Meier curves and log-rank analyses. Finally, a Cox proportional hazards model was implemented to evaluate the link between the DeRitis ratio and the main outcome, presenting results as hazard ratios (HRs) adjusted for pertinent covariates.

### Propensity score matching

In order to reduce the chances of selection bias, this research utilized PSM. Propensity scores were calculated using a binary logistic regression model, which included the following variables: age, gender, body mass index (BMI), systolic blood pressure (SBP), estimated glomerular filtration rate (eGFR), serum creatinine, blood urea nitrogen, triglycerides, total cholesterol, low-density lipoprotein (LDL), high-density lipoprotein (HDL), blood glucose levels, hemoglobin, counts of white blood cells, red blood cells, platelets, history of hypertension, history of diabetes, percutaneous coronary intervention (PCI) performed during the hospital stay, existence of complex coronary lesions (characterized by at least one of these: long lesion, restenotic lesion, small vessel disease, bifurcation lesion, chronic total occlusion, calcified lesion, or pontoon lesion), antiplatelet therapy during the hospital stay (including aspirin, clopidogrel, or ticagrelor), and use of angiotensin-converting enzyme inhibitors or angiotensin receptor blockers (ACEIs/ARBs) during the hospital stay. These covariates were selected to balance demographics, cardiovascular risk profiles, renal and hepatic function surrogates, and in-hospital treatment strategies between the groups. Matching was performed using a nearest-neighbor algorithm with a 1:1 ratio and a caliper width based on the logit of the propensity score. This process yielded two well-balanced groups: 2,840 patients with a DeRitis ratio ≥1 were successfully matched to 2,840 controls with a DeRitis ratio <1. The statistical analysis software R 4.0.5 and R package “MatchIt” were used for the matching.

## Result

### Patient characteristics

This analysis incorporated 8,429 patients diagnosed with ACS from the OPT-CAD database. Prior to PSM, 2,972 patients had a DeRitis ratio <1, and 5,457 had a DeRitis ratio ≥1. There were notable discrepancies in the initial characteristics of the two groups. Patients in the high DeRitis ratio (≥1) group were generally older and had a lower BMI, lower left ventricular ejection fraction (LVEF), and HDL levels, along with LDL levels. They also exhibited poorer renal function, as reflected in relevant biomarkers, and lower counts of red blood cells, white blood cells, and platelets. Furthermore, the high-ratio group had a lower proportion of smokers, fewer patients with a history of diabetes or prior PCI, and a higher percentage of female participants. Notably, there were no major differences observed between the two groups regarding the frequency of in-hospital PCI or the use of dual antiplatelet therapy (DAPT).

### Patient characteristics after matching

Following PSM, a total of 5,680 patients were included in the final analysis, comprising 2,840 patients with a DeRitis ratio <1 and 2,840 patients with a DeRitis ratio ≥1. Post-matching baseline characteristics were well-balanced between the two groups. During the 5-year follow-up period, the primary endpoint of NACE occurred in 806 patients. Secondary endpoints included 363 cases of all-cause death, 155 cases of non-fatal myocardial infarction, 217 strokes, and 305 major bleeding events. The mean DeRitis ratio in the high-ratio group (≥1) was 2.14, with corresponding mean AST and ALT values of 70.28 U/L and 28.31 U/L, respectively. In the low-ratio group (<1), the mean DeRitis ratio was 0.71, with mean AST and ALT values of 30.51 U/L and 47.34 U/L, respectively (Table 1).

**Table 1:** Baseline clinical characteristics.

Variables	Before PSM			After PSM		
	DeRitis <1	DeRitis ≥1		DeRitis <1	DeRitis ≥1	
	*n* = 2,972	*n* = 5,457	*P*	*n* = 2,840	*n* = 2,840	*P*
Age	59.78 ± 9.95	62.98 ± 11.08	<0.001	60.20 ± 9.80	60.23 ± 10.61	0.91
Male (%)	2,293 (77.2)	3,752 (68.8)	<0.001	2,167 (76.3)	2,160 (76.1)	0.852
Height (cm)	168.66 ± 6.78	167.05 ± 7.27	<0.001	168.54 ± 6.84	168.25 ± 6.90	0.113
BMI	24.76 ± 2.84	24.20 ± 2.92	<0.001	24.71 ± 2.82	24.62 ± 2.90	0.271
Systolic pressure (mmHg)	134.82 ± 20.28	133.97 ± 22.27	0.086	134.84 ± 20.34	135.12 ± 22.11	0.627
Smoke (%)	1,557 (52.4)	2,565 (47.0)	<0.001	1,461 (51.4)	1,465 (51.6)	0.937
Hypertension (%)	1,796 (60.4)	3,259 (59.7)	0.541	1,716 (60.4)	1,699 (59.8)	0.665
Diabetes (%)	738 (24.8)	1,224 (22.4)	0.014	710 (25.0)	710 (25.0)	1
History of stroke (%)	206 (6.9)	427 (7.8)	0.149	199 (7.0)	213 (7.5)	0.506
Previous PCI (%)	508 (17.1)	799 (14.6)	0.003	488 (17.2)	460 (16.2)	0.337
OMI (%)	258 (8.7)	416 (7.6)	0.095	249 (8.8)	250 (8.8)	1
eGFR	114.28 ± 39.33	107.16 ± 38.75	<0.001	113.65 ± 38.67	112.82 ± 39.21	0.425
LVEF (%)	60.92 ± 9.01	59.64 ± 8.97	<0.001	60.87 ± 8.99	60.36 ± 8.70	0.028
Creatinine (μmol/L)	73.86 ± 33.51	76.78 ± 33.28	<0.001	73.87 ± 32.39	74.40 ± 31.71	0.537
Blood urea nitrogen (mmol/L)	5.56 ± 2.08	5.73 ± 2.30	0.001	5.56 ± 2.07	5.54 ± 2.05	0.727
Triglycerides (mmol/L)	1.93 ± 1.35	1.77 ± 1.23	<0.001	1.91 ± 1.33	1.88 ± 1.36	0.433
Total cholesterol (mmol/L)	4.29 ± 1.13	4.50 ± 1.17	<0.001	4.31 ± 1.14	4.31 ± 1.04	0.867
Low-density lipoprotein (mmol/L)	2.50 ± 1.04	2.64 ± 1.02	<0.001	2.50 ± 0.98	2.50 ± 0.90	0.858
High-density lipoprotein (mmol/L)	1.03 ± 0.33	1.11 ± 0.33	<0.001	1.04 ± 0.34	1.03 ± 0.29	0.763
Blood glucose (mmol/L)	6.40 ± 2.53	6.58 ± 2.73	0.003	6.43 ± 2.56	6.39 ± 2.53	0.58
Hemoglobin (g/L)	137.23 ± 15.67	133.82 ± 17.12	<0.001	136.81 ± 15.66	136.51 ± 16.42	0.474
White blood cells (10^9^/L)	7.94 ± 2.63	8.47 ± 3.13	<0.001	7.98 ± 2.66	7.91 ± 2.74	0.36
Red blood cells (10^12^/L)	4.44 ± 0.53	4.35 ± 0.61	<0.001	4.44 ± 0.54	4.43 ± 0.59	0.906
Platelets (10^9^/L)	210.95 ± 57.74	207.21 ± 58.35	0.005	210.19 ± 57.47	210.56 ± 59.48	0.81
Hematocrit value (vol%)	40.33 ± 4.35	39.62 ± 4.75	<0.001	40.23 ± 4.28	40.20 ± 4.65	0.767
DeRitis ratio	0.71 ± 0.18	2.34 ± 3.26	<0.001	0.71 ± 0.18	2.14 ± 3.25	<0.001
ALT (u/L)	47.58 ± 47.72	30.73 ± 37.77	<0.001	47.34 ± 47.77	28.31 ± 31.84	<0.001
AST (u/L)	30.43 ± 28.01	83.44 ± 160.71	<0.001	30.51 ± 28.08	70.28 ± 145.61	<0.001
PCI in hospital (%)	2,096 (70.5)	3,910 (71.7)	0.286	2,010 (70.8)	2,029 (71.4)	0.598
Complex lesions (%)	1,276 (42.9)	2,369 (43.4)	0.689	1,224 (43.1)	1,234 (43.5)	0.81
DAPT in hospital (%)	2,740 (92.2)	4,964 (91.0)	0.06	2,614 (92.0)	2,594 (91.3)	0.361
Aspirin in hospital (%)	2,803 (94.3)	5,141 (94.2)	0.883	2,676 (94.2)	2,683 (94.5)	0.73
Clopidogrel in hospital (%)	2,762 (92.9)	5,009 (91.8)	0.068	2,636 (92.8)	2,618 (92.2)	0.392
Ticagrelor in hospital (%)	7 (0.2)	25 (0.5)	0.161	7 (0.2)	17 (0.6)	0.066
ACEI/ARB (%)	2,249 (75.7)	4,117 (75.4)	0.836	2,153 (75.8)	2,147 (75.6)	0.877
Aspirin at discharge (%)	2,908 (97.8)	5,341 (97.9)	0.996	2,779 (97.9)	2,790 (98.2)	0.338
Clopidogrel at discharge (%)	2,637 (88.7)	4,910 (90.0)	0.08	2,522 (88.8)	2,538 (89.4)	0.523
Ticagrelor at discharge (%)	3 (0.1)	26 (0.5)	0.009	3 (0.1)	18 (0.6)	0.002

PCI, percutaneous coronary intervention; OMI, old myocardial infarction; LVEF, left ventricular ejection fraction; ACEI/ARB, angiotensin-converting enzyme inhibitor/angiotensin receptor blocker; ALT, alanine aminotransferase; AST, aspartate aminotransferase; DAPT, dual antiplatelet therapy; DeRitis ratio was defined as AST divided ALT; complex lesion was defined as having one or more of these factors: long disease, restenosis disease, small vessel disease, bifurcation disease, chronic total occlusion, calcification of the lesion, or pontoon disease.

### DeRitis ratio and clinical endpoints

**Figure 2:**
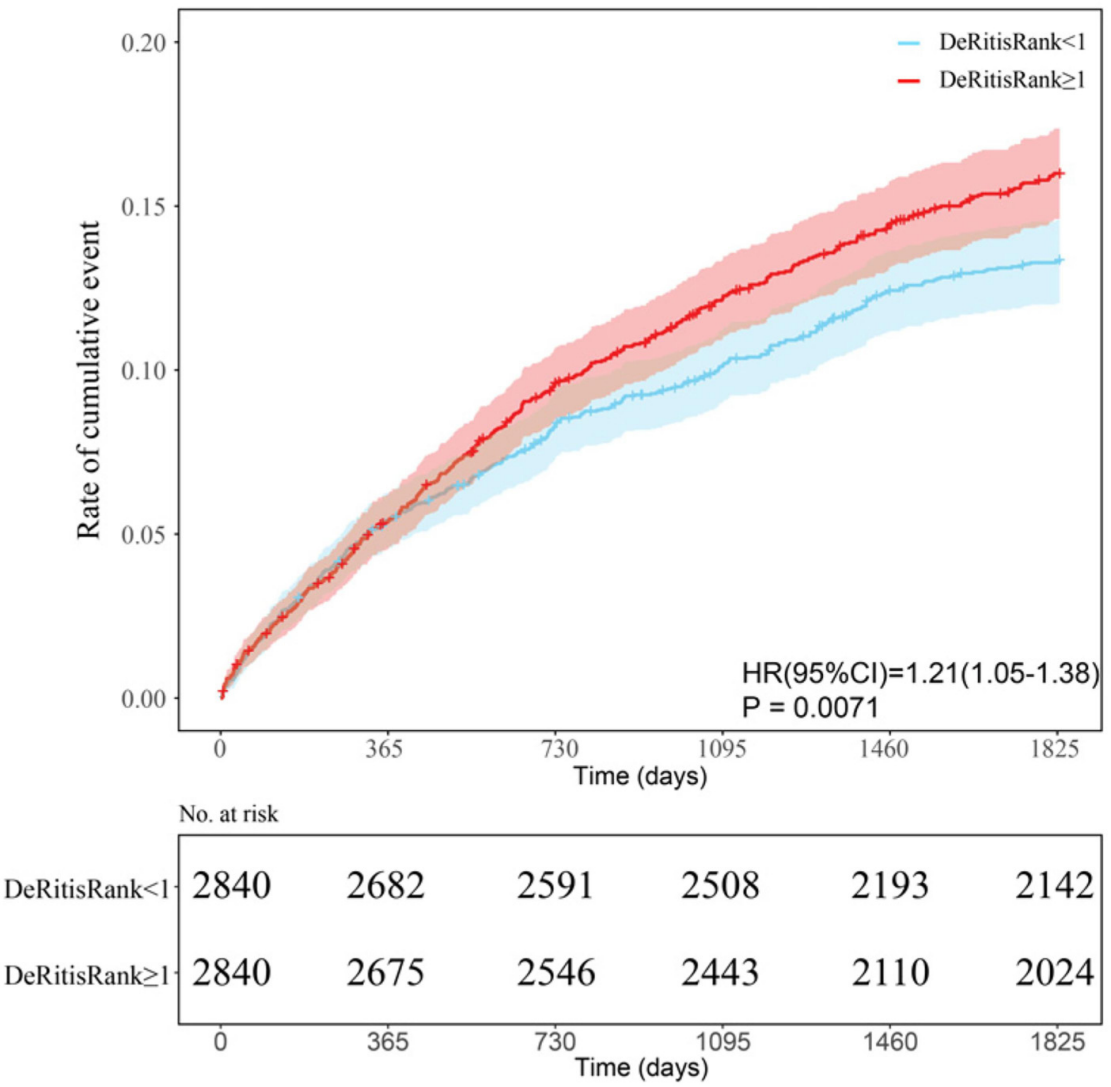
Kaplan–Meier curves for NACE within 5 years after discharge of the DeRitis ratio <1 group (blue) vs. the DeRitis ratio ≥1 group (red). HR, hazard ratio; CI, confidence interval; No. at risk, number at risk; DeRitis ratio was defined as AST divided ALT.

**Figure 3:**
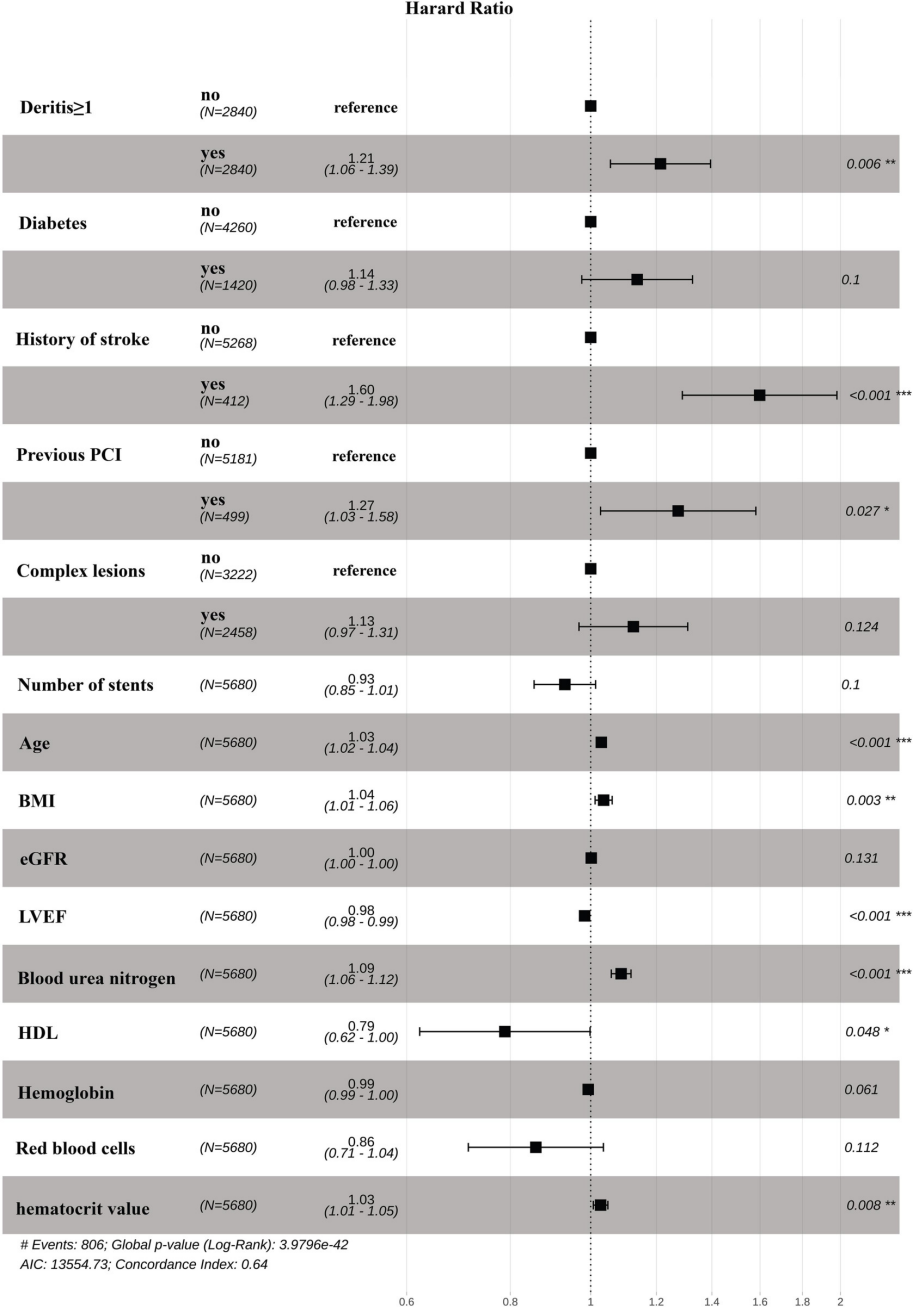
Forest plots of multivariable cox regression for NACE within 5 years after discharge. PCI, percutaneous coronary intervention; HDL, High Density Lipoprotein; LVEF, Left Ventricular Ejection Fraction; DeRitis ratio was defined as AST divided ALT; complex lesion was defined as having one or more of these factors: long disease, restenosis disease, small vessel disease, bifurcation disease, chronic total occlusion, calcification of the lesion, or pontoon disease.

Before PSM, the cumulative incidence of NACE within 5 years was 16.1% in the group with a DeRitis ratio ≥1, compared to 12.9% in the group with a DeRitis ratio <1. After PSM, the incidence remained higher in the high-ratio group (15.4%) than in the low-ratio group (13.0%) (Table 2). Kaplan–Meier analysis demonstrated a significantly higher cumulative incidence of NACE over the 5-year follow-up period in the DeRitis ratio ≥1 group compared to the <1 group (HR: 1.21; 95% CI, 1.05–1.38; *P* = 0.0071) (Figure 2). After adjustment for relevant covariates, Cox regression analysis continued to show a significantly increased risk of NACE within 5 years after discharge among patients with a DeRitis ratio ≥1 (adjusted HR, 1.21; 95% CI, 1.06–1.39; *P* = 0.006) (Figure 3).

**Table 2:** Follow-up results at 5 years after discharge.

Variables	Before PSM			After PSM		
	DeRitis <1	DeRitis ≥1		DeRitis <1	DeRitis ≥1	
	*n* = 2,972	*n* = 5,457	*P*	*n* = 2,840	*n* = 2,840	*P*
NACE (%)	382 (12.9)	877 (16.1)	<0.001	368 (13.0)	438 (15.4)	0.009
All-cause death (%)	167 (5.6)	453 (8.3)	<0.001	164 (5.8)	199 (7.0)	0.065
Non-fatal MI (%)	75 (2.5)	176 (3.2)	0.081	70 (2.5)	85 (3.0)	0.254
Stroke (%)	113 (3.8)	211 (3.9)	0.93	108 (3.8)	109 (3.8)	1
Major bleeding (%)	139 (4.7)	307 (5.6)	0.071	135 (4.8)	170 (6.0)	0.045

NACE, net adverse clinical event; major bleeding was defined as BARC type 2–5 bleeding within 5 years after discharge.

**Figure 4:**
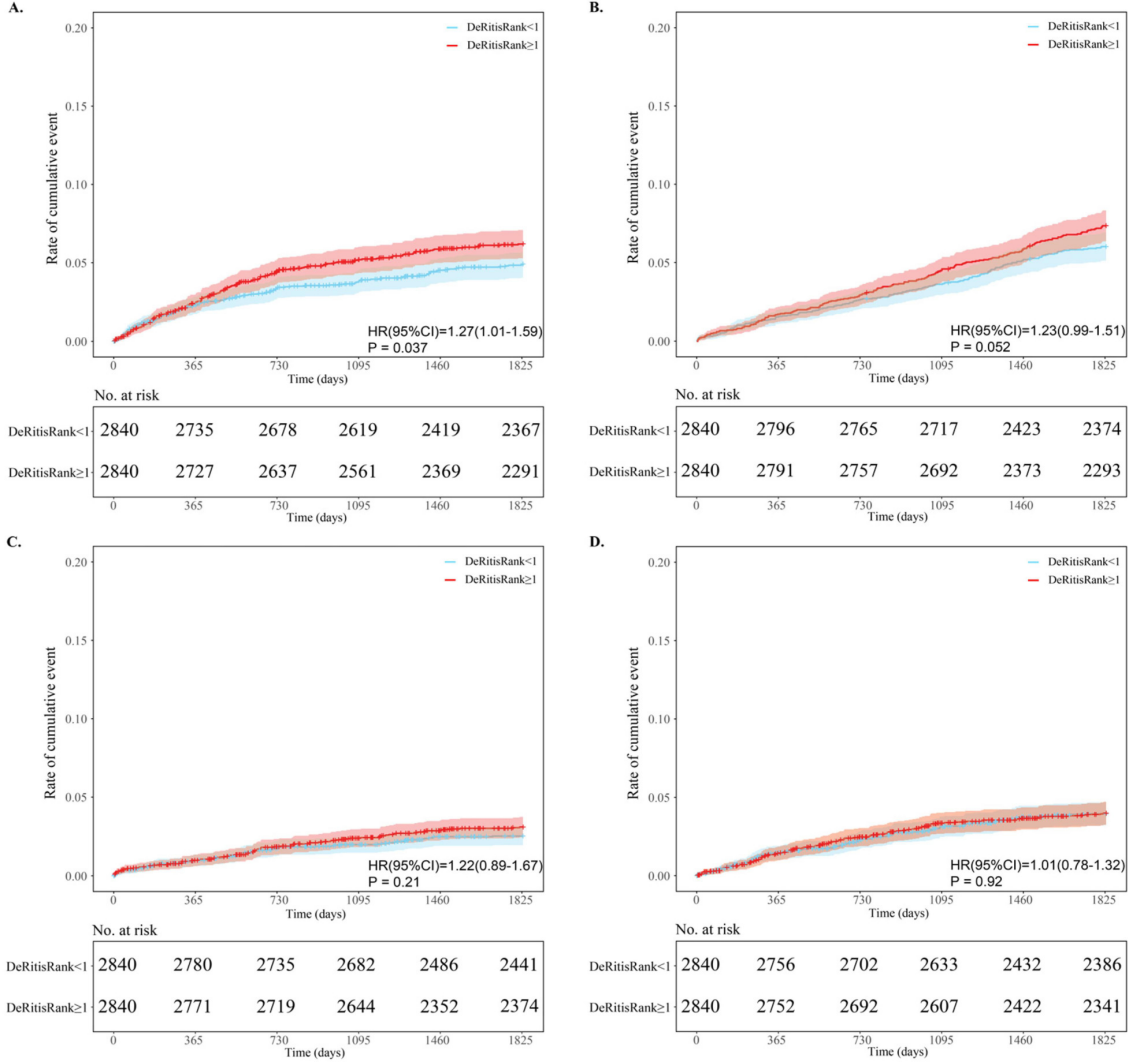
Kaplan–Meier curves for major bleeding **(A)**, all-cause death **(B)**, non-fatal MI **(C)** and stroke **(D)** of the DeRitis ratio <1 group (blue) vs. the DeRitis ratio ≥1 group (red). HR, hazard ratio; CI, confidence interval; No. at risk, number at risk; DeRitis ratio was defined as AST divided ALT.

Before PSM, the incidence of all-cause death was significantly higher in the DeRitis ratio ≥1 group compared to the <1 group (8.3% vs. 5.6%, *P* < 0.001). After matching, the difference in all-cause mortality, although still numerically higher in the high-ratio group (7.0% vs. 5.8%), was no longer statistically significant (*P* = 0.065). Conversely, no significant difference in major bleeding was observed before matching (5.6% vs. 4.7%, *P* = 0.071), but the incidence became significantly higher in the high-ratio group after PSM (6.0% vs. 4.8%, *P* = 0.045). No significant differences were found between the two groups in the incidence of non-fatal MI or stroke, either before or after matching (Table 2). Kaplan–Meier analysis of secondary endpoints further indicated that patients with a DeRitis ratio ≥1 had a 1.27-fold higher risk of major bleeding than those with a ratio <1 (HR: 1.27; 95% CI: 1.01–1.59; *P* = 0.037) (Figure 4A). Although the cumulative risk of all-cause death showed a trend toward separation over the 5-year follow-up, this did not reach statistical significance (HR: 1.23; 95% CI: 0.99–1.51; *P* = 0.052) (Figure 4B). No significant differences were observed in the cumulative risks of non-fatal MI (HR: 1.22; 95% CI: 0.89–1.67; *P* = 0.21) or stroke (HR: 1.01; 95% CI: 0.78–1.32; *P* = 0.92) between the two groups (Figures 4C,D).

### Risk factors for NACE at 5 years after discharge

To identify independent risk factors associated with NACE within 5 years after discharge, a Cox proportional hazards model was constructed using stepwise variable selection. The final multivariable model is summarized in Figure 3.

Several factors were significantly associated with an increased risk of NACE: history of stroke (HR: 1.60; 95% CI: 1.29–1.98; *P* < 0.001), previous PCI (HR: 1.27; 95% CI: 1.03–1.58; *P* = 0.027), advancing age (per year increase) (HR: 1.03; 95% CI: 1.02–1.04; *P* < 0.001), higher BMI (HR: 1.04; 95% CI: 1.01–1.06; *P* = 0.003), elevated blood urea nitrogen (HR: 1.09; 95% CI: 1.06–1.12; *P* < 0.001), and higher hematocrit (HR: 1.03; 95% CI: 1.01–1.05; *P* = 0.008). Protective factors included a higher LVEF (HR: 0.98; 95% CI: 0.98–0.99; *P* < 0.001) and a higher HDL level (HR: 0.79; 95% CI: 0.62–1.00; *P* = 0.048).

The model also incorporated additional variables, including history of diabetes, presence of multivessel disease, number of stents, red blood cell count, hemoglobin, and estimated eGFR, though these did not reach statistical significance.

## Discussion

In recent years, an increasing number of serum biomarkers initially established in non-cardiac fields have been repurposed to predict adverse outcomes in CHD (19, 20). The DeRitis ratio, initially introduced to assess hepatocyte damage and later adopted as a prognostic marker in oncology (21, 22), has garnered interest in cardiovascular research. However, its connection to long-term negative outcomes in ACS patients remains uncertain. Our study demonstrates that ACS patients with a DeRitis ratio ≥1 faced a markedly increased risk of NACE within 5 years after discharge, even after adjustment for multiple potential confounders, including history of diabetes or stroke, prior PCI, presence of multivessel disease, number of stents implanted, age, BMI, eGFR, LVEF, blood urea nitrogen, HDL level, hemoglobin, red blood cell count, and hematocrit. Further analysis revealed that the disparity in prognosis was largely due to a higher occurrence of major bleeding among patients with an elevated DeRitis ratio.

While there was no statistically significant variance in survival rates between the two groups, patients with a DeRitis ratio ≥1 showed a higher numerical mortality rate than those whose ratio <1. This trend may be attributed to poorer baseline liver function in the high-ratio group, which aligns with the established association between an elevated DeRitis ratio and impaired liver health (3). Furthermore, the Kaplan–Meier curves for major bleeding began to diverge after one year post-discharge, with relatively flat trajectories observed during the initial 12-month period. This delayed separation suggests that patients with a higher DeRitis ratio may be at increased long-term risk of bleeding, possibly due to reduced hepatic metabolic capacity affecting the clearance of antiplatelet medications. Consequently, such patients might benefit from earlier discontinuation of aspirin rather than prolonged monotherapy after the period of DAPT. However, it's crucial to acknowledge that some studies have reported a protective effect of long-term aspirin use on liver function. Thus, the optimal antiplatelet strategy in this patient subgroup remains uncertain and warrants further investigation.

AST and ALT are routinely measured in the diagnostic workup of CHD. ALT is primarily synthesized in the liver, and elevated ALT levels are a specific indicator of hepatocellular injury. In contrast, AST is released by various tissues, including the liver, heart, skeletal muscle, and red blood cells (23). Although an elevation in AST may reflect myocardial damage, its lack of organ specificity limits its utility as a standalone prognostic marker in CHD (24). The pathogenesis of CHD involves not only localized cardiac damage but also systemic alterations affecting multiple organs and pathways. Thus, while AST alone may lack specificity for cardiac injury, it may provide insight into the overall systemic status of patients with ACS. Potential mechanisms linking AST to cardiovascular prognosis include systemic inflammation, insulin resistance, oxidative stress, and underlying metabolic syndrome (10). Given these considerations, the DeRitis ratio —integrating both enzymes—is likely a more robust and clinically relevant biomarker than either enzyme alone for predicting adverse outcomes in CHD. It reflects not only hepatic function but also broader systemic metabolic and inflammatory processes, offering a more holistic assessment of patient risk.

A study conducted within a healthy East Asian population aimed to identify novel cardiovascular risk factors using the DeRitis ratio. It concluded that elevated AST/ALT ratios correlated with a reduced risk of cardiovascular risk (15)—a finding that contrasts with our results. This disparity could be due to variations in study populations: the prior research involved healthy individuals, whereas our investigation focused specifically on patients diagnosed with ACS. Furthermore, the endpoint in the earlier study was limited to ischemic events, while our study employed a more comprehensive endpoint, NACE, which incorporates both ischemic and bleeding events. It is noteworthy that multiple studies have reported associations between elevated DeRitis ratios and conditions such as metabolic syndrome and insulin resistance (11, 19). These are recognized risk factors for unfavorable cardiovascular results. These associations indirectly support the notion that a high DeRitis ratio may reflect a systemic metabolic disturbance conducive to adverse clinical events, particularly in high-risk groups like those with ACS.

Based on our understanding, this study is the initial exploration of the link between the DeRitis ratio and long-term negative clinical outcomes in patients with ACS. Our findings provide novel evidence supporting the potential role of the DeRitis ratio in evaluating risk and prognosis for ACS patients. However, several limitations should be acknowledged. First, although this analysis utilized data from the prospective OPT-CAD registry, the present study was retrospective in design. Despite comprehensive adjustment for known confounders through multivariable regression and PSM, residual bias from unmeasured confounding factors may still influence the results. Second, several important potential confounders were not systematically collected in the OPT-CAD database and thus could not be adjusted for. These include specific etiologies of liver enzyme elevation (such as viral hepatitis B or C serology, detailed alcohol use history, and severe tricuspid insufficiency as assessed by echocardiography), procedural details (such as radial vs. femoral access, operator experience, and off-hours procedures), periprocedural complications (such as coronary artery perforation, which is associated with complex interventions and worse immediate outcome s^25^), and detailed antithrombotic regimens for patients with atrial fibrillation (including specific anticoagulant type and dose). The lack of data on bioresorbable vascular scaffolds (BVS) or bare-metal stents (BMS) reflects their minimal use in the study period in China, where second-generation drug-eluting stents predominated. Contemporary studies from other regions have explored BVS use in ACS (26), highlighting the evolving interventional landscape during our enrollment period. Similarly, prasugrel was not available in China during the enrollment period. The absence of these granular data introduces the possibility of residual confounding, particularly regarding bleeding risk. For instance, inappropriate dosing of anticoagulants in atrial fibrillation patients is known to impact bleeding and thrombotic outcomes (27). Future prospective studies designed to validate the DeRitis ratio should incorporate these important clinical variables. Third, AST and ALT measurements were obtained only during the initial hospitalization; sequential measurements throughout the follow-up period were unavailable. Therefore, the influence of dynamic changes in liver enzyme levels or the DeRitis ratio over time on clinical outcomes remains unexplored. Additional extensive and forward-looking research is necessary to confirm the prognostic value of the DeRitis ratio and clarify its clinical utility in Chinese ACS patients.

## Conclusion

The DeRitis ratio is significantly associated with the long-term risk of NACE after hospital discharge in ACS patients. A DeRitis ratio ≥1 serves as a distinct factor for NACE, with affected patients experiencing a 1.21-fold higher risk of events within 5 years after discharge compared to those with a ratio <1. Additionally, an elevated DeRitis ratio is correlated with a greater occurrence of major bleeding episodes. These findings from an observational study warrant further prospective validation to assess their potential utility in clinical risk stratification.

## Data Availability

The raw data supporting the conclusions of this article will be made available by the authors, without undue reservation.
